# Epicardial fat area as an independent predictor of atrial fibrillation occurrence and severity

**DOI:** 10.3389/fcvm.2026.1797765

**Published:** 2026-05-21

**Authors:** Xiting Liu, Ani Gao, Sixu Liu, Fengzhi Sun, Xin Shi

**Affiliations:** Department of Cardiovascular Medicine, Affiliated Zhongshan Hospital of Dalian University, Dalian, Liaoning, China

**Keywords:** atrial fibrillation, epicardial adipose tissue, epicardial fat area, left atrial volume index, obesity

## Abstract

**Objective:**

This study aimed to investigate the relationship between the epicardial fat area (EFA), and both the occurrence and severity of atrial fibrillation (AF), and explore the potential factors associated with AF development.

**Methods:**

Three hundred hospitalized patients with AF (100 paroxysmal, 100 persistent, and 100 permanent) and 200 patients with sinus rhythm (SR), who were admitted during the same period, were retrospectively included. The EFA was quantified using chest computed tomography images analyzed with the sliceOmatic software. Then, the demographic, laboratory, echocardiographic, and EFA data were collected and compared between groups. Afterwards, logistic regression and receiver-operating characteristic (ROC) analyses were performed to identify the factors associated with AF.

**Results:**

The body mass index, neutrophil-to-lymphocyte ratio, red blood cell distribution width, blood urea nitrogen, serum creatinine, blood glucose, left ventricular end-diastolic diameter, and left atrial volume index (LAVI) were higher in AF patients, when compared with SR controls (all, *p* < 0.05). The EFA was significantly elevated in AF patients [13.42 [9.06–17.72] *vs.* 7.30 [5.29–9.32], *p* < 0.001], with a progressive increase from paroxysmal to persistent, to permanent AF. The multivariate analysis identified EFA (odds ratio: 1.307, 95% confidence interval: 1.214–1.407) and LAVI (odds ratio: 1.057, 95% confidence interval: 1.034–1.081) as independent factors associated with AF occurrence. In the AF subgroups, EFA and LAVI were also identified as independent factors associated with AF severity. Furthermore, age, body mass index, red blood cell distribution width, serum creatinine, and LAVI were identified as independent determinants of EFA. The ROC analyses revealed good predictive performance for AF, with EFA showing an area under the ROC curve of 0.836 (cut-off: 10.08 cm^2^, sensitivity: 69.30%, specificity: 83.00%) and LAVI showing an area under the ROC curve of 0.812 (cut-off: 30.82 mL/m^2^, sensitivity: 74.00%, specificity: 80.50%).

**Conclusion:**

EFA is closely associated with both the occurrence and severity of AF. Along with LAVI, EFA may serve as a valuable independent factor associated with the risk of AF.

## Introduction

Atrial fibrillation (AF) is the most common sustained cardiac arrhythmia, causing impaired left atrial contraction, thrombus formation, and potentially, severe thromboembolic events (1, 2). Globally, approximately 37.5 million individuals are affected, representing 0.5% of the population (3). Its prevalence and severity increase with age, reaching 5%–9% in individuals between 60 and 80 years old, and over 10% in individuals above 80 years old (4). Comorbidities, such as ischemic heart disease and heart failure, which are more frequent in older adults, further elevate AF risk (4). AF may present with palpitations, chest discomfort, dyspnea, or dizziness. However, a number of patients remain asymptomatic, and symptomatic episodes often occur unnoticed (5). Consequently, early diagnosis, timely management, and prevention remain as critical challenges in cardiology.

Obesity is strongly associated with AF, and its prevalence in AF patients is increasing worldwide (6). Epidemiological data have shown that obesity significantly raises AF risk, in which each 1-unit increase in body mass index (BMI) is associated with a 6% increase in AF incidence (7). A large-scale study that involved over 9.7 million individuals who underwent national health examinations reported that overweight and obese patients have higher risks of new-onset AF, when compared with normal-weight controls (8). Similarly, a population-based longitudinal study conducted in Stockholm revealed that a one standard deviation increase in BMI is associated with higher AF risk [odds ratio (OR): 1.25, 95% confidence interval (CI): 1.12–1.40] (9). Consistently, animal models further support the link between obesity and AF, in which weight reduction was shown to decrease AF susceptibility (10).

Recent evidence suggests that regional or visceral fat may play a more critical role in AF development, when compared to overall obesity (11). Epicardial adipose tissue (EAT), which is a visceral fat depot located between the myocardial surface and visceral pericardium, is closely associated with obesity (12). EAT consists primarily of adipocytes, but also contains nerves, ganglia, inflammatory and immune cells, and stromal vessels (13). It is metabolically active, and functions as an endocrine organ, secreting cytokines and inflammatory mediators, including interleukins, tumor necrosis factor, resistin, monocyte chemoattractant protein, adiponectin, and angiotensinogen, via paracrine and endocrine pathways (14). These factors promote local inflammation and oxidative stress, creating a substrate for AF (15). Observational and meta-analysis studies have revealed that AF patients have higher EAT volumes, and EAT has been identified as an independent risk factor for AF (16, 17).

Epicardial fat volume (EFV) has traditionally been used to quantify EAT. A previous meta-analytic study indicated that EFV is higher in AF patients, when compared to healthy subjects, with persistent AF patients having greater EFV, when compared to paroxysmal AF patients (18). However, EFV measurement is time-consuming and labor-intensive. The epicardial fat area (EFA) offers a simpler and faster alternative for estimating EAT, showing a strong correlation with EFV (*r* = 0.77–0.92, *p* < 0.001) and comparable diagnostic performance (19). These findings suggest that EFA can reliably represent EAT quantity, and may serve as a useful predictor for cardiovascular risk.

Despite the increasing recognition of EAT in AF pathophysiology, limited evidence exists on the association between EFA, and both AF occurrence and severity. The present study aimed to investigate the relationship between EFA and AF, and evaluate its association with AF occurrence and progression.

## Methods

### Study design

The present study was designed as a retrospective case-control analysis, which included patients with AF as the case group and patients with sinus rhythm (SR) as the control group. In addition, subgroup analyses were performed for AF patients to determine the factors associated with AF severity.

### Participants and enrollment criteria

A total of 300 patients with AF (100 paroxysmal, 100 persistent, and 100 permanent) and 200 patients with SR, who were hospitalized at Zhongshan Hospital, Dalian University, between January 2022 and December 2023, were retrospectively included. The baseline clinical data, which included the demographic information, medical history, laboratory tests, and imaging findings, were collected from the hospital electronic medical record system for analysis. The study protocol was approved by the Ethics Committee of Zhongshan Hospital, Dalian University (Approval no. KY2023-134-1).

The inclusion criteria were, as follows (1): confirmed diagnosis of AF based on the Chinese Society of Cardiology Guidelines for the Diagnosis and Treatment of Atrial Fibrillation (2023) (https://pubmed.ncbi.nlm.nih.gov/37312479/); (2) availability of complete clinical records, laboratory results, and imaging data; (3) voluntary consent to participate in the study.

The exclusion criteria were, as follows: (1) patients with severe structural heart diseases, including valvular disease (moderate-to-severe stenosis or regurgitation), acute myocardial infarction, rheumatic heart disease, congenital heart disease, cardiomyopathy, or myocarditis; (2) patients with a history of cardiac surgery (e.g., coronary artery bypass grafting, valve replacement, and radiofrequency ablation); (3) patients with advanced heart failure [New York Heart Association (NYHA ) class IV]; (4) patients with active infections or inflammatory diseases; (5) patients with severe hepatic or renal dysfunction, thyroid disease, malignancy, hematological disorders, or autoimmune diseases; (6) patients with incomplete clinical records.

The control group consisted of 200 patients who underwent routine non-contrast chest computed tomography (CT) during the same period, and met the following criteria: sinus rhythm confirmed by electrocardiography (ECG), no history of AF, and the absence of severe structural heart disease or NYHA class IV heart failure. These patients were admitted for various clinical indications, including health check-ups, non-cardiovascular conditions, and mild cardiovascular symptoms, without evidence of significant structural abnormalities.

### Demographic and clinical data

The baseline demographic and clinical characteristics were collected, which included age, sex, height, weight, systolic blood pressure (SBP), diastolic blood pressure (DBP), history of hypertension, and history of diabetes. The laboratory results were also retrieved, which included white blood cell count (WBC), neutrophil count, lymphocyte count, red blood cell distribution width (RDW), total protein (TP), alanine aminotransferase (ALT), aspartate aminotransferase (AST), blood urea nitrogen (BUN), serum creatinine (Scr), serum uric acid (SUA), fasting glucose (GLU), triglycerides (TG), total cholesterol (TC), high-density lipoprotein cholesterol (HDL-C), low-density lipoprotein cholesterol (LDL-C), lipoprotein(a) [Lp(a)], and D-dimer. BMI was calculated as weight (kg) divided by height squared (m^2^).

### Echocardiography

All patients underwent transthoracic echocardiography. The measurements included the left ventricular end-diastolic diameter (LVDD), left ventricular ejection fraction (LVEF), and three left atrial dimensions (anteroposterior, transverse, and superior-inferior diameters). The left atrial volume index (LAVI, mL/m^2^) was calculated, as follows:

$$\mathrm{LAVI}=\frac{\mathrm{left}\;\mathrm{atrial}\;\mathrm{volume}(\mathrm{LAV},\;\mathrm{mL})}{\mathrm{body}\;\mathrm{surface}\;\mathrm{area}\;(\mathrm{BSA},\;m^{2})}$$

Where LAV was estimated, as follows:

$$\begin{array}{rl} \mathrm{LAV}(\mathrm{mL})= & \;4\pi /3\times \mathrm{anteroposterior}\;\mathrm{diameter}\;(\mathrm{cm})\;/\;2\\ & \;\times \;\mathrm{transverse}\;\mathrm{diameter}\;(\mathrm{cm})\;/\;2\;\\ & \;\times \;\mathrm{superior}\text{-}\mathrm{inferior}\;\mathrm{diameter}\;(\mathrm{cm})\;/\;2 \end{array}$$

and BSA was calculated using the following formula:

$$\begin{array}{rl} \mathrm{BSA}(m^{2})= & \;0.0061\times \mathrm{height}\;(\mathrm{cm})+0.0128\\ & \times \mathrm{weight}(\mathrm{kg})-0.1529 \end{array}$$

### Measurement of EFA

All patients underwent routine non-contrast chest CT using the Siemens Somatom Definition AS 64-slice CT scanner (Siemens Healthineers, Forchheim, Germany). The scans were performed during a single end-inspiratory breath-hold, with the patient in the supine position and arms raised above the head. The scanning range extended from the thoracic inlet to the costophrenic angles. The acquisition parameters were, as follows: tube voltage, 100–120 kV; tube current, 200–280 mA; slice thickness, 5 mm; slice interval, 5 mm; pitch, 1.0; matrix, 512 × 512; field of view, 500 × 500 mm; collimation, 64 × 0.625 mm; rotation time, 0.5 s per rotation. After raw data acquisition, the images were reconstructed using a bone algorithm with a slice thickness and interval of 1 mm for further analysis.

**Figure 1:**
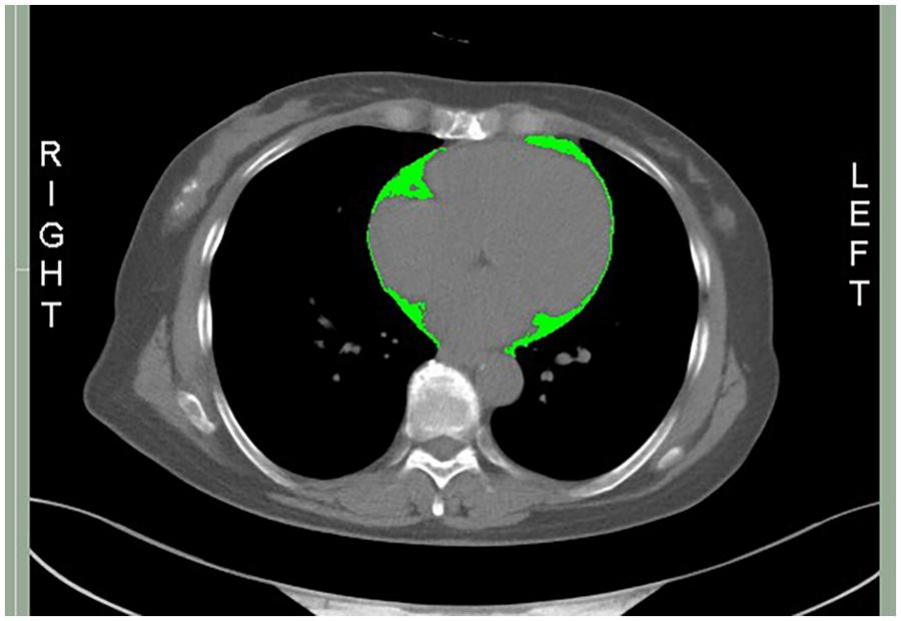
Epicardial adipose tissue. The epicardial fat area (EFA, highlighted in green) was quantified using chest computed tomography (CT) images.

EFA was quantified using the chest CT images analyzed with the sliceOmatic software (version 5.0; Tomo vision, Montreal, QC, Canada). The attenuation threshold for adipose tissue was defined as −190 to −30 Hounsfield units (HU) (20–23). The measurement region extended from the pulmonary valve superiorly to the diaphragm inferiorly. At the mid-ventricular level, the epicardial fat contour was manually delineated by two independent observers blinded to the clinical data. Then, the software automatically calculated the EFA (cm^2^) based on the outlined region (highlighted in green, Figure 1). In the present study, EFA represented as a two-dimensional measurement (cm^2^) derived from a single mid-ventricular CT slice, rather than a three-dimensional volumetric assessment.

### Statistical analysis

All statistical analyses were performed using IBM SPSS Statistics version 23.0. Categorical variables were expressed in counts and percentages, and compared using Chi-square test. Continuous variables were initially tested for normality using the Shapiro–Wilk test. For normally distributed data, two-group comparisons were performed using independent-samples *t*-test, and the results were presented in mean ± standard deviation (SD). Comparisons among three groups were conducted using one-way analysis of variance (ANOVA), with pairwise comparisons performed using the least significant difference (LSD) test. For non-normally distributed data, two-group comparisons were analyzed using the Wilcoxon rank-sum test, and three-group comparisons were performed using the Kruskal–Wallis *H*-test, with Bonferroni correction for pairwise comparisons. These data were expressed in median (P25, P75).

Analyses of factors associated with AF were conducted using univariate and multivariate logistic regression. Associations between EFA and related clinical parameters were evaluated using Spearman correlation and linear regression analyses. Variables with *p* < 0.10 in the univariate analysis were entered into the multivariate model using a forward stepwise selection approach, with an entry criterion of *p* < 0.10 and a retention criterion of *p* < 0.05. EFA was found to be significantly correlated with BMI, RDW, and Scr (all, *p* < 0.001). In order to reduce the potential impact of multicollinearity, forward stepwise selection was used to construct the final multivariate logistic regression model. Variables that did not meet the criteria for inclusion were excluded from the final model. Associations between EFA and related clinical parameters were further evaluated using Spearman correlation and linear regression analyses. Receiver operating characteristic (ROC) curve analysis was applied to assess the predictive value of factors associated with AF. In order to externally validate the discriminative performance of EFA and LAVI for AF, an independent validation cohort of 80 participants was recruited from the same center. This cohort included 20 controls and 60 patients with AF, which comprised of 20 patients with paroxysmal AF, 20 patients with persistent AF, and 20 patients with permanent AF. The ROC curve analysis was repeated in the validation cohort to assess the predictive value of EFA and LAVI.

A two-tailed *p*-value of <0.05 was considered statistically significant.

## Results

### Comparison of baseline characteristics and cardiac parameters between SR and AF patients

The present study included 200 patients in the SR group (mean age: 69.71 ± 9.75 years old, 51.50% male) and 300 patients in the AF group (mean age: 70.98 ± 9.37 years old, 55.67% male). As shown in Table 1, there were no significant differences between groups, in terms of age, sex, prevalence of hypertension or diabetes, SBP, DBP, WBC, TP, ALT, AST, SUA, TG, TC, HDL-C, LDL-C, Lp(a), or D-dimer (all, *p* > 0.05). However, AF patients had significantly higher BMI, NLR, RDW, BUN, Scr and GLU, when compared with the SR group (all, *p* < 0.05).

**Table 1:** Demographic and clinical characteristics of the SR and AF groups.

Characteristic	SR group (*n* = 200)	AF group (*n* = 300)	*p*-value
Age (years)	69.71 ± 9.75	70.98 ± 9.37	0.143
Male (%)	103 (51.50)	167 (55.67)	0.360
Female (%)	97 (48.50)	133 (44.33)	
Diabetes (*n*, %)	26 (13.0%)	52 (17.3%)	0.191
Hypertension (*n*, %)	42 (21.0%)	78 (26.0%)	0.200
SBP (mmHg)	130.00 (120.00, 140.00)	132.00 (121.00, 146.00)	0.233
DBP (mmHg)	80.00 (70.25, 90.00)	80.00 (72.00, 90.00)	0.827
BMI (kg/m^2^)	24.22 (21.97, 27.14)	25.55 (23.46, 27.78)	<0.001
WBC (×10^9^/L)	6.00 (4.96, 7.06)	5.88 (5.02, 7.14)	0.736
NLR (%)	2.17 (1.67, 3.33)	2.43 (1.85, 3.67)	0.021
RDW (%)	42.50 (41.03, 44.10)	44.30 (42.43, 45.90)	<0.001
TB (g/L)	68.00 (64.53, 72.30)	67.30 (63.10, 71.70)	0.114
ALT (U/L)	19.50 (15.00, 28.00)	20.00 (14.00, 26.00)	0.597
AST (U/L)	23.00 (19.00, 28.00)	23.00 (20.00, 29.00)	0.430
BUN (mmol/L)	5.53 (4.72, 6.53)	6.06 (5.00, 7.59)	<0.001
Scr (*μ*mol/L)	66.35 (54.08, 75.68)	69.55 (57.88, 81.78)	0.007
SUA (μmol/L)	324.85 (274.58, 398.75)	340.00 (274.00, 411.38)	0.352
GLU (mmol/L)	5.14 (4.77, 5.63)	5.46 (4.92, 6.43)	<0.001
TG (mmol/L)	1.31 (1.00, 1.69)	1.20 (0.97, 1.60)	0.099
TC (mmol/L)	4.52 ± 0.99	4.30 ± 1.05	0.278
HDL-C (mmol/L)	1.18 (0.99, 1.42)	1.17 (0.99, 1.36)	0.369
LDL-C (mmol/L)	2.55 (1.94, 3.15)	2.69 (2.02, 3.33)	0.070
Lp(a) (mg/dL)	96.00 (46.15, 207.24)	115.23 (59.96, 220.17)	0.067
D-dimer (ng/mL)	0.20 (0.15, 0.34)	0.21 (0.14, 0.32)	0.560

SR, sinus rhythm; AF, atrial fibrillation; SBP, systolic blood pressure; DBP, diastolic blood pressure; BMI, body mass index; WBC, white blood cell count; NLR, neutrophil-to-lymphocyte ratio; RDW, red blood cell distribution width; TB, total protein; ALT, alanine aminotransferase; AST, aspartate aminotransferase; BUN, blood urea nitrogen; SUA, serum uric acid; Scr, serum creatinine; GLU, blood glucose; TG, triglycerides; TC, total cholesterol; HDL-C, high-density lipoprotein cholesterol; LDL-C, low-density lipoprotein cholesterol; Lp(a), lipoprotein(a).

The echocardiographic results revealed that AF patients had significantly larger LVDD and LAVI, when compared with the SR group (*p* < 0.05), while LVEF did not significantly differ between the two groups (*p* > 0.05) (Table 2). In addition, AF patients had significantly greater EFA, when compared with patients in the SR group [13.42 [9.06, 17.72] *vs.* 7.30 [5.29, 9.32], *p* < 0.001].

**Table 2:** Echocardiographic indexes of the SR and AF groups.

Index	SR group (*n* = 200)	AF group (*n* = 300)	*p*-value
LVDD (mm)	46.00 (44.00, 48.00)	47.00 (45.00, 50.00)	0.001
LVEF (%)	62.00 (60.00, 65.00)	63.00 (58.00, 66.00)	0.819
LAVI (mL/m^2^)	22.77 (18.15, 29.39)	38.60 (30.45, 52.73)	<0.001

SR, sinus rhythm; AF, atrial fibrillation; LVDD, left ventricular end-diastolic diameter; LVEF, left ventricular ejection fraction; LAVI, left atrial volume index.

### Logistic regression analysis of factors associated with AF development

In order to identify the potential factors associated with AF development, univariate logistic regression was performed on variables that exhibited significant differences between groups. EFA, BMI, RDW, Scr, GLU, LVDD and LAVI had OR values above 1, indicating a positive correlation with AF risk (*p* < 0.05). These were considered factors associated with AF development. However, NLR and BUN were not significantly associated with AF (*p* > 0.05) (Table 3).

**Table 3:** Univariate logistic regression analysis of AF occurrence.

Variable	Regression coefficient	Standard error	Wald *X^2^*	*p*-value	OR	95% CI
EFA	0.347	0.034	103.784	<0.001	1.414	(1.323–1.512)
BMI	0.074	0.023	10.035	0.002	1.076	(1.028–1.126)
NLR	0.021	0.030	0.482	0.487	1.021	(0.963–1.083)
RDW	0.116	0.030	15.226	<0.001	1.123	(1.059–1.190)
BUN	0.064	0.040	2.476	0.116	1.066	(0.984–1.006)
Scr	0.011	0.004	6.661	0.010	1.011	(1.003–1.020)
GLU	0.163	0.063	6.733	0.009	1.177	(1.041–1.331)
LVDD	0.077	0.022	12.211	<0.001	1.080	(1.034–1.127)
LAVI	0.093	0.010	85.606	<0.001	1.098	(1.076–1.119)

AF, atrial fibrillation; OR, odds ratio; CI, confidence interval; EFA, epicardial fat area; BMI, body mass index; NLR, neutrophil-to-lymphocyte ratio; RDW, red blood cell distribution width; BUN, blood urea nitrogen; Scr: serum creatinine; GLU, blood glucose; LVDD, left ventricular end-diastolic diameter; LAVI, left atrial volume index.

Variables identified in the univariate analysis were included in the multivariate logistic regression to control for potential confounding effects. The analysis results revealed that EFA and LAVI were independently associated with AF (*p* < 0.001). Therefore, these were identified as independent factors associated with AF development (Table 4).

**Table 4:** Multivariate logistic regression analysis of AF occurrence.

Variable	Regression coefficient	Standard error	Wald *X^2^*	*p*-value	OR	95% CI
EFA	0.267	0.038	50.586	<0.001	1.307	(1.214–1.407)
LAVI	0.055	0.011	24.281	<0.001	1.057	(1.034–1.081)

AF, atrial fibrillation; OR, odds ratio; CI, confidence interval; EFA, epicardial fat area; LAVI, left atrial volume index.

### Comparison of baseline characteristics and cardiac parameters among AF subtypes

The present study included 100 patients in each AF subtype group: paroxysmal AF (mean age: 69.81 ± 8.46 years old, 47.0% male), persistent AF (mean age: 70.69 ± 9.97 years old, 60.0% male), and permanent AF (mean age: 72.44 ± 9.52 years old, 60.0% male). No significant differences were observed among the AF subtypes, in terms of age, sex, prevalence of hypertension or diabetes, SBP, or DBP (all, *p* > 0.05). BMI significantly differed across the groups (*p* = 0.04) (Table 5). However, no significant differences were observed among the three AF subgroups for WBC, NLR, TP, ALT, AST, TG, TC, GLU, HDL-C, LDL-C, Lp(a), or D-dimer (all, *p* > 0.05), while RDW, BUN, Scr and SUA significantly differed across the groups (*p* < 0.05) (Table 5).

**Table 5:** Demographic and clinical characteristics of the AF subgroups.

Characteristic	Paroxysmal AF (*n* = 100)	Persistent AF (*n* = 100)	Permanent AF (*n* = 100)	*p*-value
Age (years)	69.81 ± 8.46	70.69 ± 9.97	72.44 ± 9.52	0.100
Male (%)	47 (47.0%)	60 (60.0%)	60 (60.0%)	0.102
Female (%)	53 (53.0%)	40 (40.0%)	40 (40.0%)	
Diabetes (*n*, %)	28 (28.0%)	25 (25.0%)	30 (30.0%)	0.729
Hypertension (*n*, %)	18 (18.0%)	17 (17.0%)	21 (21.0%)	0.752
SBP (mmHg)	131.00 (120.00, 146.50)	129.00 (120.00, 143.59)	138.00 (123.00, 150.00)	0.112
DBP (mmHg)	80.00 (81.00, 85.75)	80.00 (73.00, 90.00)	80.00 (72.50, 90.00)	0.227
BMI (kg/m^2^)	24.86 (23.03, 27.46)	25.86 (24.23, 27.77)	26.07 (23.68, 29.41)	0.040
WBC (×10^9^/L)	6.05 (5.04, 7.14)	6.16 (5.14, 7.32)	5.72 (4.98, 7.09)	0.595
NLR (%)	2.33 (1.85, 3.36)	2.47 (1.80, 3.82)	2.61 (1.91, 3.96)	0.482
RDW (%)	43.20 (41.40, 44.58)	44.3 (43.10, 46.00)	45.00 (43.13, 47.08)	<0.001
TB (g/L)	67.03 ± 5.83	67.62 ± 6.89	67.26 ± 6.21	0.805
ALT (U/L)	19.00 (15.00, 25.00)	19.50 (15.00, 29.75)	20.00 (14.00, 26.00)	0.796
AST (U/L)	22.50 (20.00, 27.00)	24.00 (20.00, 30.00)	23.00 (20.00, 52.00)	0.239
BUN (mmol/L)	5.73 (4.74, 7.09)	6.32 (5.02, 7.50)	6.41 (5.26, 8.08)	0.019
Scr (μmol/L)	66.95 (56.68, 79.85)	67.20 (56.28, 81.13)	74.00 (60.78, 89.33)	0.014
SUA (μmol/L)	319.04 ± 87.85	351.07 ± 114.67	383.99 ± 118.89	<0.001
GLU (mmol/L)	5.38 (4.81, 6.41)	5.41 (4.92, 6.33)	5.58 (4.99, 6.49)	0.487
TG (mmol/L)	1.32 (1.01, 1.62)	1.17 (0.97, 1.61)	1.17 (0.90, 1.56)	0.227
TC (mmol/L)	4.44 ± 1.07	4.44 ± 1.06	4.16 ± 0.99	0.150
HDL-C (mmol/L)	1.21 (1.03, 1.39)	1.18 (0.98, 1.38)	1.14 (0.97, 1.31)	0.376
LDL-C (mmol/L)	2.65 ± 0.89	2.74 ± 0.85	2.58 ± 0.93	0.910
Lp(a) (mg/dL)	118.00 (65.03, 228.62)	99.23 (61.58, 196.88)	120.85 (58.89, 224.96)	0.640
D-dimer (ng/mL)	0.19 (0.13, 0.30)	0.19 (0.14, 0.31)	0.24 (0.17, 0.34)	0.080

AF, atrial fibrillation; SBP, systolic blood pressure; DBP, diastolic blood pressure; BMI, body mass index; WBC, white blood cell count; NLR, neutrophil-to-lymphocyte ratio; RDW, red blood cell distribution width; TB, total protein; ALT, alanine aminotransferase; AST, aspartate aminotransferase; BUN, blood urea nitrogen; SUA, serum uric acid; Scr, serum creatinine; GLU, blood glucose; TG, triglycerides; TC, total cholesterol; HDL-C, high-density lipoprotein cholesterol; LDL-C, low-density lipoprotein cholesterol; Lp(a), lipoprotein(a).

The echocardiographic measurements revealed significant differences among the AF subtypes, in terms of LVDD, LVEF and LAVI (*p* < 0.05) (Table 6). Furthermore, EFA significantly differed among the subtypes [paroxysmal AF: 8.78 (7.31, 10.96), persistent AF: 13.50 (10.75, 16.52), permanent AF: 17.96 (15.24, 21.54); *p* < 0.001].

**Table 6:** Echocardiographic indexes of the AF subgroups.

Index	Paroxysmal AF	Persistent AF	Permanent AF	*p*-value
LVDD (mm)	46.00 (44.25, 48.00)	47.00 (46.00, 49.75)	47.00 (45.25, 51.00)	0.003
LVEF (%)	64.00 (61.00, 67.00)	60.50 (58.00, 66.00)	62.00 (58.00, 66.00)	0.001
LAVI (mL/m^2^)	26.62 (19.89, 32.89)	45.48 (35.74, 53.71)	50.34 (37.89, 64.29)	<0.001

AF, atrial fibrillation; LVDD, left ventricular end-diastolic diameter; LVEF, left ventricular ejection fraction; LAVI, left atrial volume index.

Overall, EFA, RDW, BUN, Scr, SUA, LVDD, LVEF and LAVI significantly differed among the AF subtypes (*p* < 0.05). The pairwise comparisons revealed that EFA (all, *p* < 0.001) and SUA (*p* < 0.05) significantly differed between all group pairs, increasing from paroxysmal to persistent, to permanent AF.

### Logistic regression analysis of factors associated with AF severity

In order to identify the factors associated with AF severity, variables with significant differences among the three subtypes were analyzed using univariate logistic regression, with paroxysmal AF as the reference group. The univariate analysis results revealed that EFA, RDW, SUA and LAVI were positively associated with AF severity, in both the persistent and permanent AF subgroups (all, OR > 1, *p* < 0.05). In contrast, LVEF was negatively associated with AF severity (OR < 1, *p* < 0.05). Furthermore, the associations of BMI, BUN, Scr and LVDD with AF severity were inconsistent across the two subgroups (Table 7).

**Table 7:** Univariate logistic regression analysis of AF severity.

Variable	Persistent AF			Permanent AF		
	Regression coefficient	*p*-value	OR	Regression coefficient	*p*-value	OR
EFA	0.327	<0.001	1.387	0.573	<0.001	1.774
BMI	0.058	0.095	9.337	0.098	0.005	1.103
RDW	0.157	0.001	1.169	0.189	<0.001	1.207
BUN	0.071	0.268	1.073	0.139	0.022	1.149
Scr	0.004	0.458	1.004	0.013	0.022	1.013
SUA	0.003	0.026	1.003	0.006	<0.001	1.006
LVDD	0.052	0.134	1.053	0.123	<0.001	1.130
LVEF	−0.087	0.015	0.916	−0.094	<0.001	0.911
LAVI	0.125	<0.001	1.133	1.136	<0.001	1.391

AF, atrial fibrillation; OR, odds ratio; EFA, epicardial fat area; BMI, body mass index; RDW, red blood cell distribution width; BUN, blood urea nitrogen; Scr, serum creatinine; SUA, serum uric acid; LVDD, left ventricular end-diastolic diameter; LVEF, left ventricular ejection fraction; LAVI, left atrial volume index.

Variables identified in the univariate analysis were included in the multivariate logistic regression to adjust for potential confounders. EFA and LAVI were independently associated with AF severity (*p* < 0.001), serving as independent factors (Table 8).

**Table 8:** Multivariate logistic regression analysis of AF severity.

Variable	Persistent AF			Permanent AF		
	Regression coefficient	*p*-value	OR	Regression coefficient	*p*-value	OR
EFA	0.261	<0.001	1.298	0.092	<0.001	1.655
LAVI	0.094	<0.001	1.098	0.092	<0.001	1.097

AF, atrial fibrillation; OR, odds ratio; EFA, epicardial fat area; LAVI, left atrial volume index.

### Correlation of EFA with clinical parameters and predictive value for AF

EFA was identified as an independent factor associated with AF occurrence and severity. The spearman correlation analysis revealed significant positive correlations between EFA, and age (*p* = 0.009), DBP (*p* = 0.023), BMI (*p* < 0.001), NLR (*p* = 0.004), RDW (*p* < 0.001), BUN (*p* < 0.001), Scr (*p* < 0.001), SUA (*p* = 0.001), GLU (*p* = 0.04), LVDD (*p* < 0.001), and LAVI (*p* < 0.001), and significant negative correlations with HDL-C (*p* = 0.04) and LVEF (*p* = 0.005).

In order to identify the independent determinants of EFA, variables significantly correlated with EFA in the Spearman analysis were included in the linear regression model. Age (*p* = 0.011), BMI (*p* < 0.001), RDW (*p* = 0.002), Scr (*p* = 0.029), and LAVI (*p* < 0.001) were identified as independently associated with EFA.

**Figure 2:**
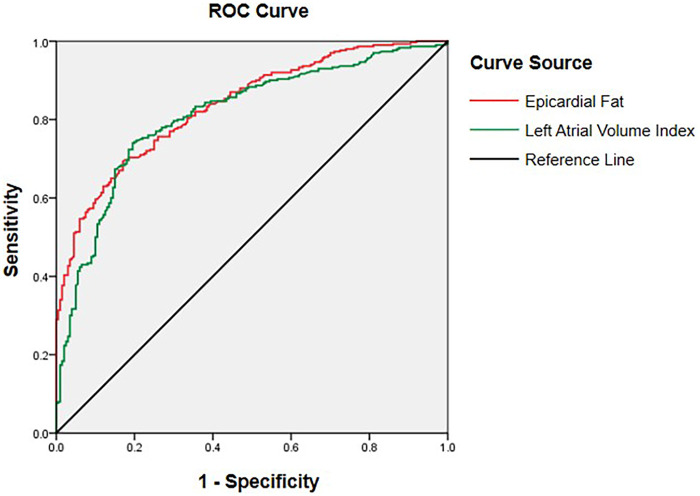
Receiver operating characteristic (ROC) curve analysis of the epicardial fat area (EFA) and left atrial volume index (LAVI) for predicting atrial fibrillation (AF) occurrence.

Lastly, the ROC curve analysis results revealed that both EFA and LAVI had good predictive value for AF occurrence (Figure 2). The optimal cut-off value for EFA was 10.08 (cm^2^), with an area under the receiver operating characteristic curve (AUC) of 0.836 (95% CI: 0.802–0.870), a sensitivity of 69.3%, and a specificity of 83.0%. For LAVI, the cut-off was 30.82 (mL/m^2^), with an AUC of 0.812 (95% CI: 0.773–0.850), a sensitivity of 74.0%, and a specificity of 80.5%.

External validation was performed in an independent cohort of 80 participants, which included 20 controls and 60 patients with AF (20 paroxysmal, 20 persistent, and 20 permanent AF). The results are presented in Supplementary Table S1 and Supplementary Figure S1. For EFA, the AUC for discriminating AF was 0.909 (95% CI: 0.845–0.972). The optimal cut-off value was 10.70 mm^2^, yielding a sensitivity of 0.700 and a specificity of 0.950. For LAVI, the AUC was 0.881 (95% CI: 0.805–0.957). The optimal cut-off value was 28.40 (mL/m^2^), with a sensitivity of 0.817 and a specificity of 0.950. These findings were highly consistent with those observed in the derivation cohort (EFA AUC = 0.836; LAVI AUC = 0.812), suggesting that both EFA and LAVI have good discriminative performance for AF across different patient groups, without evidence of substantial overfitting.

## Discussion

In the present retrospective case-control study, it was found that EFA is closely associated with both the occurrence and severity of AF. Several clinical and echocardiographic parameters, such as BMI, RDW, Scr, GLU, LVDD and LAVI, were identified as potential factors associated with AF. The further multivariate analyses results revealed that EFA and LAVI are independent factors associated with both AF occurrence and severity. In addition, the ROC curve analysis confirmed that these two indices provide good discriminatory ability for identifying patients at risk of AF. Collectively, these findings highlight the clinical relevance of EFA as a simple imaging marker in improving AF risk stratification.

EAT plays an important physiological role in supporting cardiac function. Under normal conditions, it provides metabolic support by absorbing excess free fatty acids during ischemia, generates heat to maintain myocardial temperature, offers mechanical protection, and exerts endocrine functions by secreting adipokines (24). However, in obesity, excessive accumulation of EAT is associated with myocardial hypertrophy, fibrosis, and apoptosis, accompanied by reduced adiponectin secretion, and increased production of pro-inflammatory mediators (25). EAT expansion is considered the hallmark of visceral obesity, and directly contributes to the development of obesity-related cardiovascular complications, including coronary artery disease, heart failure, and AF (26, 27). Recent evidence has highlighted the concept of atrial cardiomyopathy, which encompasses the structural, electrical, and functional remodeling of the atria, and may serve as a key intermediary that links epicardial adipose tissue to the pathogenesis of AF (28). Among the various factors secreted by EAT, adiponectin is of particular importance. Although adiponectin normally exerts anti-inflammatory and anti-fibrotic effects, such as inhibiting tumor necrosis factor alpha-mediated endothelial activation via prostacyclin induction, and suppressing inflammatory signaling through protein kinase A- and AMP-activated protein kinase-dependent pathways (29), its levels are markedly reduced in pathological states (30). Hypoadiponectinemia is frequently observed in obesity and metabolic syndrome, which are conditions strongly linked to increased risk of AF (31). In addition, EAT can promote both local and systemic chronic inflammation, which further contributes to AF pathogenesis (32). Present evidence suggests that the arrhythmogenic potential of EAT is largely mediated through inflammatory pathways. Specifically, EAT-derived cytokines may trigger localized myocardial inflammation, leading to the generation of C-reactive protein (33), which in turn generates metabolites that impair sodium-calcium exchange, disrupt membrane integrity, and induce intracellular calcium overload (34). These changes can reduce calcium channel activity, shorten atrial action potential duration and refractory periods, and facilitate premature atrial excitation, thereby initiating and sustaining AF. In the present study, EFA was used as the quantitative indicator of EAT. It was found that EFA is closely associated with both the occurrence and severity of AF.

The relationship between left atrial (LA) size and AF has been increasingly recognized. LA enlargement is associated with higher incidence of AF, and tends to progress as AF becomes more persistent (35). A positive correlation has been observed between EAT and LA diameter, suggesting that EAT accumulation may exert mechanical stress on the myocardium, increase ventricular mass, impair left ventricular function, and promote cardiac remodeling, thereby facilitating AF development (36). A multivariable regression analysis identified LA size as an independent predictor of paroxysmal AF (37). The LAVI provides a more objective assessment of LA size, since it minimizes the influence of sex, age, height and weight, while exhibiting lower variability and higher reproducibility (38). LAVI has been shown to outperform LA anteroposterior diameter in predicting AF recurrence (39). Kranert et al. reported that elevated LAVI is more prevalent in patients with recurrent AF after cardioversion or catheter ablation, serving as an independent predictor (40). Similarly, Nakamori et al. evaluated participants with a history of AF and age-matched controls with other cardiovascular diseases, and found that AF patients exhibited significantly increased EAT, which correlated with the LA volume (41). These findings suggest that EAT may contribute to LA structural remodeling beyond other AF-related pathogenic factors. In the present study, it was found that both LAVI and EFA were independently associated with AF occurrence and severity, supporting its potential use in predicting AF.

Elevated RDW has been associated with increased risk of AF development. A positive correlation was found between RDW, and both AF occurrence and severity, highlighting RDW as a potential risk factor for AF (42). AF itself can trigger inflammatory responses, reduce red blood cell lifespan, and consequently increase RDW in peripheral blood counts (43). The cross-sectional analysis of 106,998 participants from a Chinese cohort revealed that elevated RDW was significantly associated with higher prevalence of AF (44). Similarly, Guan et al. reported that among the 991 AF patients and 991 matched non-AF controls, RDW levels were significantly higher in the AF group (15.09 ± 1.93 *vs.* 14.89 ± 1.91, *p* = 0.017) (45). However, RDW exhibits limited diagnostic value for AF, with a ROC analysis yielding an optimal cut-off of 14.1 (AUC = 0.526, sensitivity = 65.8%, specificity = 39.5%) (45). Mechanistically, alterations in red blood cell size can reduce erythrocyte deformability and increase blood viscosity, impair microcirculatory flow, promote myocardial ischemia, and facilitate the structural and electrical remodeling of the atria, ultimately predisposing to AF (46). Consistent with these findings, the investigators observed that RDW was significantly higher in AF patients, when compared with SR controls, and was positively correlated with EFA.

Elevated blood glucose and diabetes have been identified as risk factors for the development of AF (47). In the hyperglycemic state, atrial myocytes convert excess glucose into glycogen with each contraction. Glycogen accumulation within the intercellular space can disrupt normal conduction, alter gap junction proteins between atrial myocytes, and impair electrical coupling, thereby promoting arrhythmogenesis, and increasing susceptibility to AF (48). A meta-analysis of 34 studies revealed the dose-response relationship between blood glucose levels and AF risk (49). Hyperglycemia has also been linked to atrial structural remodeling. Wang et al. studied atrial tissues obtained from 86 patients who underwent coronary artery bypass grafting, and found that patients with diabetes had significantly larger left atrial diameters and LAVI, when compared with non-diabetic controls (*p* < 0.001), as well as more pronounced atrial fibrosis (50). Iacobellis et al. measured the EAT thickness and GLU in 115 non-diabetic participants, and found that individuals with elevated GLU had significantly greater EAT thickness, with a strong positive correlation between EAT and GLU (*r* = 0.60, *p* < 0.001) (51). In the present study, GLU was positively associated with AF occurrence, indicating its potential contribution to AF risk.

### Limitations

The present study was limited by its relatively small sample size and single-center design, which may have restricted the generalizability of the findings and introduced potential bias. Larger, multicenter studies with more comprehensive analyses are needed to validate these results, and better elucidate the risk factors that influence AF onset and progression. Second, although an independent external validation cohort was included, the cohort size was relatively modest, and all participants were recruited from the same single center. The limited sample size may have resulted in relatively wide confidence intervals around the AUC estimates, and reduced statistical precision. In addition, the single-center design may have introduced selection bias. Furthermore, the relatively small number of controls in the validation cohort may have affected the stability of the specificity estimates. Therefore, larger multicenter prospective studies are needed to further validate the predictive performance of EFA and LAVI for AF. Third, the CT scans in the present study were not ECG-gated, since these were performed as part of the routine clinical chest CT, rather than a dedicated cardiac imaging. ECG-gated cardiac CT is considered the gold standard for minimizing motion artifacts in epicardial adipose tissue measurement. The absence of ECG-gating may have introduced some degree of measurement variability, which can affect the accuracy of EFA quantification. Future studies that utilize ECG-gated protocols are warranted to validate the present findings. Fourth, as a retrospective hospital-based study, the detailed admission diagnoses for the control subjects were not systematically available. Although strict exclusion criteria were applied to minimize confounding, some control patients may have had underlying conditions that can influence systemic inflammation, and potentially affect the EFA measurements. Future prospective studies with well-defined community-based controls are warranted to validate the present findings. In addition, EFA was measured from a single mid-ventricular slice on routine non-contrast, non-ECG-gated chest CT, rather than from a dedicated ECG-gated cardiac CT or three-dimensional epicardial fat volume assessment. Although this approach is more practical for routine clinical use, it may be susceptible to motion artifacts and variability in slice positioning. Fifth, several clinically relevant covariates that may influence both AF and EAT, such as AF duration, antihypertensive and lipid-lowering therapy, RAAS blockers, SGLT2 inhibitors, GLP-1 receptor agonists, heart failure severity, and sleep apnea, were not available in the present retrospective study. Therefore, residual confounding from these unmeasured factors cannot be excluded. Future prospective studies with more comprehensive data collection are needed to confirm the present findings. Sixth, due to incomplete records from the original data analysis, precise correlation coefficients for the Spearman correlations were not available. Although this limits the precise assessment of correlation strengths, the significance of these relationships was demonstrated by the reported *p*-values. Furthermore, the finding that EFA is an independent factor associated with AF occurrence and severity was robustly validated in the multivariate regression analyses. Seventh, the present study quantified epicardial fat using a single-slice area, rather than a three-dimensional volume. Although the feasibility of this method has been validated in previous studies, and revealed significant associations with AF occurrence and severity in the present analysis, three-dimensional volumetry provides a more comprehensive assessment of global epicardial fat burden (19). This represents a limitation of the present study. Future research using cardiac CT or magnetic resonance imaging (MRI) with volumetric quantification is warranted to validate the present findings. Lastly, the present study did not assess the EAT CT attenuation values, which have recently been recognized as potential imaging biomarkers that reflect local adipose tissue inflammation and fibrotic remodeling (52, 53). Recent evidence has suggested that EAT density may be independently associated with AF recurrence and severity, providing information beyond the EAT volume or area (54). The absence of CT attenuation data represents a limitation of the present analysis. Thus, future prospective studies that combine the EAT area, density, and inflammatory biomarkers are needed to fully characterize the role of EAT in AF pathogenesis.

## Conclusion

The present study identified EFA as an independent factor associated with AF. This not only reflects the severity of the disease, but also demonstrates its potential in predicting AF occurrence. These findings support the potential for the wider clinical application of EFA, offering a valuable tool for risk stratification, guiding interventions, and informing strategies to prevent AF onset and recurrence.

## Data Availability

The raw data supporting the conclusions of this article will be made available by the authors, without undue reservation.

## Supplementary material

Supplementary Figure S1 ROC curves of EFA and LAVI for AF occurrence in the external validation cohort.
