# Supplementary figures and images for: Epicardial fat area as an independent predictor of atrial fibrillation occurrence and severity
Source: Front Cardiovasc Med. 2026 May 21;13:1797765. doi: 10.3389/fcvm.2026.1797765 (PMC13233377; doi:10.3389/fcvm.2026.1797765)

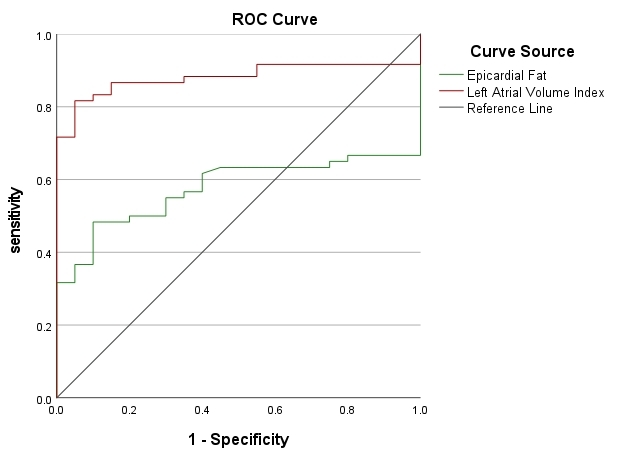

Supplement: Supplementary Figure S1 — ROC curves of EFA and LAVI for AF occurrence in the external validation cohort. [file Image1.jpeg]
